# Regenerative extracellular vesicles reshape systemic aging: lessons from antler stem cells

**DOI:** 10.7150/ijbs.140033

**Published:** 2026-08-24

**Authors:** Seung-Jae Lee, Jaehoon Bae, Ye Ji Cheon, Eun-Kyung Kim

**Affiliations:** 1 Functional Biomaterial Research Center, Korea Research Institute of Bioscience and Biotechnology, Jeongeup, 56212, Korea.; 2 Department of Applied Biotechnology, University of Science and Technology (UST), Daejeon, 34113, Korea.; 3 Functional Food Research Institute, Industry-university Cooperation Foundation, Daegu-hanny University, Gyeongsan 38610, Korea.; 4 Department of Food Industry, Daegu Haany University, Gyeongsan, 38610, Korea.; 5 Department of Marine Bio Food Science, Chonnam National University, Yeosu 59626, Korea.; 6 Nutritional Education Major, Graduate School of Education, Dong-A University, Busan 49315, Korea.

Aging is increasingly recognized as a systemic process characterized by declining regenerative capacity, disrupted intercellular communication, chronic inflammation, and progressive loss of tissue homeostasis 2,6,7. Although geroprotective interventions such as senolytics, caloric-restriction mimetics, and partial cellular reprogramming have advanced rapidly, achieving coordinated functional improvement across multiple organs remains a major challenge. In a recent study published in Nature Aging, Hao and colleagues demonstrated that extracellular vesicles derived from antler blastema progenitor cells (ABPC-EVs) attenuated multiple aging-associated phenotypes in aged mice and rhesus macaques 1. Rather than demonstrating complete reversal of organismal aging, these findings suggest that selected features of biological aging remain amenable to modulation through regenerative intercellular signaling.

Deer antlers are among the few mammalian organs capable of complete and repeated regeneration during adulthood. This remarkable regenerative capacity is sustained by antler blastema progenitor cells (ABPCs), highly proliferative cells responsible for the annual formation of vascularized bone, cartilage, connective tissue, and associated structures, distinguishing them from conventional bone marrow-derived mesenchymal stromal cells 1. However, despite their unique biological properties, the direct clinical application of deer-derived cells is unlikely to be feasible because of cross-species immune rejection, limited control over cell fate, and manufacturing complexity. In this context, ABPC-derived extracellular vesicles (ABPC-EVs) provide an attractive translational alternative by preserving regenerative signaling without introducing living xenogeneic cells. Their ability to transfer diverse bioactive cargoes while offering opportunities for engineering and product standardization makes them a logical bridge between this unique mammalian regeneration model and translational geroscience. Nevertheless, these advantages should not be interpreted as evidence of established immunological safety. Repeated administration of deer-derived EVs may still elicit antibody production, complement activation, and other innate or adaptive immune responses, which remain to be systematically evaluated.

Systemic administration of ABPC-EVs improved bone-related measures, motor coordination, physical activity, cognitive performance, inflammatory indices, and several senescence-associated phenotypes in aged mice 1. The treatment also reduced blood DNA methylation age and attenuated aging-associated histological and molecular alterations across multiple tissues, supporting partial improvement of selected biological aging markers rather than global epigenetic rejuvenation. In rhesus macaques, ABPC-EVs were likewise associated with encouraging improvements in skeletal, locomotor, inflammatory, imaging, and biological-age-related measures 1. However, because the non-human primate study included only three animals per group, these findings should be interpreted as exploratory proof-of-concept evidence. In particular, the reported increase in brain volume should be regarded as promising preliminary neuroimaging evidence that warrants further mechanistic investigation. Likewise, the study did not demonstrate long-term disease prevention, delayed frailty, lifespan extension, persistence of treatment effects after discontinuation, or efficacy in humans (Fig. 1).

One of the most important conceptual advances of this study is the suggestion that age-related functional decline may reflect the progressive failure of regenerative communication networks. Classical frameworks emphasize accumulated molecular damage, genomic instability, telomere attrition, and loss of proteostasis 2,7, whereas impaired intercellular communication is now recognized as a fundamental hallmark of aging 7. Heterochronic parabiosis and related studies of circulating rejuvenating factors have demonstrated that a young systemic environment can restore aspects of regenerative capacity in aged tissues, whereas aged circulating signals impair tissue repair in younger organisms 4,5. Building upon this conceptual framework, ABPC-EVs suggest that regenerative signals derived from a regeneration-specialized mammalian tissue can be packaged, systemically delivered, and functionally transferred to aged recipients. The conceptual advance therefore lies not in claiming that aging has been reversed, but in suggesting that selected components of regenerative communication remain pharmacologically accessible, even in aged organisms.

The reported biological responses intersect with multiple hallmarks of aging, including cellular senescence, chronic inflammation, metabolic dysregulation, stress-response decline, and altered intercellular communication 1,7. The diversity of these responses supports a systems-level interpretation in which EV cargoes influence multiple recipient-cell programs rather than a single linear pathway. Nevertheless, biological effects observed in bone, brain, or other tissues do not by themselves establish efficient EV delivery to those sites or identify the cells that internalize the vesicles. Intravenously administered EVs commonly accumulate in the liver, spleen, and lungs, and only a limited fraction may reach less accessible target tissues. Consequently, future studies should integrate quantitative biodistribution analysis with cellular uptake mapping and tissue-specific pharmacodynamic measurements to distinguish direct cargo delivery from secondary systemic effects.

Mechanistically, multi-omic analyses revealed that ABPC-EVs carry diverse molecular cargoes linked to proteostasis, oxidative-stress resistance, telomere maintenance, tissue regeneration, and inflammatory regulation 1. Among these cargoes, Prkar2a emerged as an enriched mRNA species and contributed to anti-senescence and osteogenic responses in experimental models. Importantly, the Prkar2a transcript should be distinguished from its encoded PRKAR2A protein, a regulatory subunit of cAMP-dependent protein kinase that may influence cell survival, cell-cycle control, apoptosis, and inflammation. However, the relevant recipient cell types, the extent to which Prkar2a is translated into PRKAR2A protein, and the downstream pathways responsible for organism-wide responses remain incompletely defined. Moreover, ABPC-EVs contain diverse RNAs, proteins, and other bioactive molecules, and their multi-organ effects are therefore more likely to reflect the coordinated action of multiple EV cargoes than the activity of Prkar2a alone. Future loss-of-function, cargo-depletion, and recipient-cell-specific studies will be important to determine the necessity, sufficiency, and cooperative interactions of individual EV cargoes.

ABPC-EVs also invite comparison with partial cellular reprogramming, another emerging strategy aimed at restoring tissue function during aging 8. Although reprogramming may achieve broader cellular resetting, its translational application remains constrained by delivery challenges, genomic and epigenomic instability, loss of cell identity, and tumorigenic risk. By contrast, EV-based strategies avoid direct manipulation of the recipient genome while offering opportunities for modular engineering of cargo composition and tissue targeting. Nonetheless, descriptions of EVs as inherently safe or readily translatable should be avoided. EV composition may vary according to donor characteristics, passage number, culture conditions, stress exposure, medium composition, isolation methods, and storage conditions, making biological potency more difficult to standardize. Rather than competing strategies, EV-based therapies and partial cellular reprogramming should be regarded as mechanistically distinct yet potentially complementary approaches to regenerative medicine.

Before clinical translation can be realistically achieved, several manufacturing and regulatory challenges will need to be addressed. Robust clinical translation will depend on standardized donor-cell banks, serum-free culture systems, GMP-compatible manufacturing, scalable isolation and purification, and validated procedures for storage and distribution. Donor-to-donor and batch-to-batch variability must also be controlled through predefined quality attributes, including identity, purity, potency, sterility, and product-release criteria. The cross-species origin of ABPC-EVs introduces an additional challenge because repeated administration may induce antibody production, complement activation, altered clearance, or inflammatory responses. Routine hematological and organ-function assessments provide only preliminary evidence of tolerability. Longer-term studies should therefore evaluate thrombosis, fibrosis, chronic tissue accumulation, abnormal cell proliferation, tumor-promoting activity, and delayed immune toxicity. Addressing these challenges will ultimately determine whether native ABPC-EVs, engineered EV derivatives, or defined synthetic cargo formulations represent the most clinically feasible therapeutic strategy.

The broad spectrum of biological effects observed with ABPC-EVs is particularly relevant to age-associated disorders characterized by impaired tissue repair, chronic inflammation, and declining systemic resilience, including osteoporosis, sarcopenia, neurodegenerative disease, metabolic dysfunction, and cardiovascular disease 6. At the same time, improvements across multiple physiological systems make it more challenging to define causal mechanisms and optimal therapeutic dosing. Future studies should therefore establish dose-response relationships, treatment durability, sex- and age-dependent responses, disease-specific efficacy, and comparative performance relative to established EV platforms. Equally important will be determining whether engineering or tissue-targeting strategies can improve therapeutic precision without compromising safety, and whether robust potency assays can reliably predict in vivo efficacy across manufacturing batches. Addressing these questions will be essential for determining whether regeneration-derived EVs can ultimately be translated into reproducible therapeutic platforms rather than remaining compelling experimental observations.

Overall, the work by Hao and colleagues establishes an important conceptual bridge between the extraordinary regenerative capacity of annually renewing antlers and the systemic signaling functions of extracellular vesicles. Its principal contribution lies not only in demonstrating that ABPC-EVs attenuate multiple aging-associated phenotypes across rodent and exploratory non-human primate models, but also in proposing regenerative communication as a potentially modifiable dimension of biological aging. Although the current evidence does not demonstrate durable organismal rejuvenation or lifespan extension, it strongly justifies further investigation of regeneration-derived EVs as candidates for translational geroscience. Whether these regenerative signals can ultimately be translated into clinically meaningful interventions will depend on rigorous mechanistic validation, quantitative biodistribution analyses, long-term immunological safety, and manufacturing standards that ensure reproducibility and scalability.

## Figures and Tables

**Figure 1:**
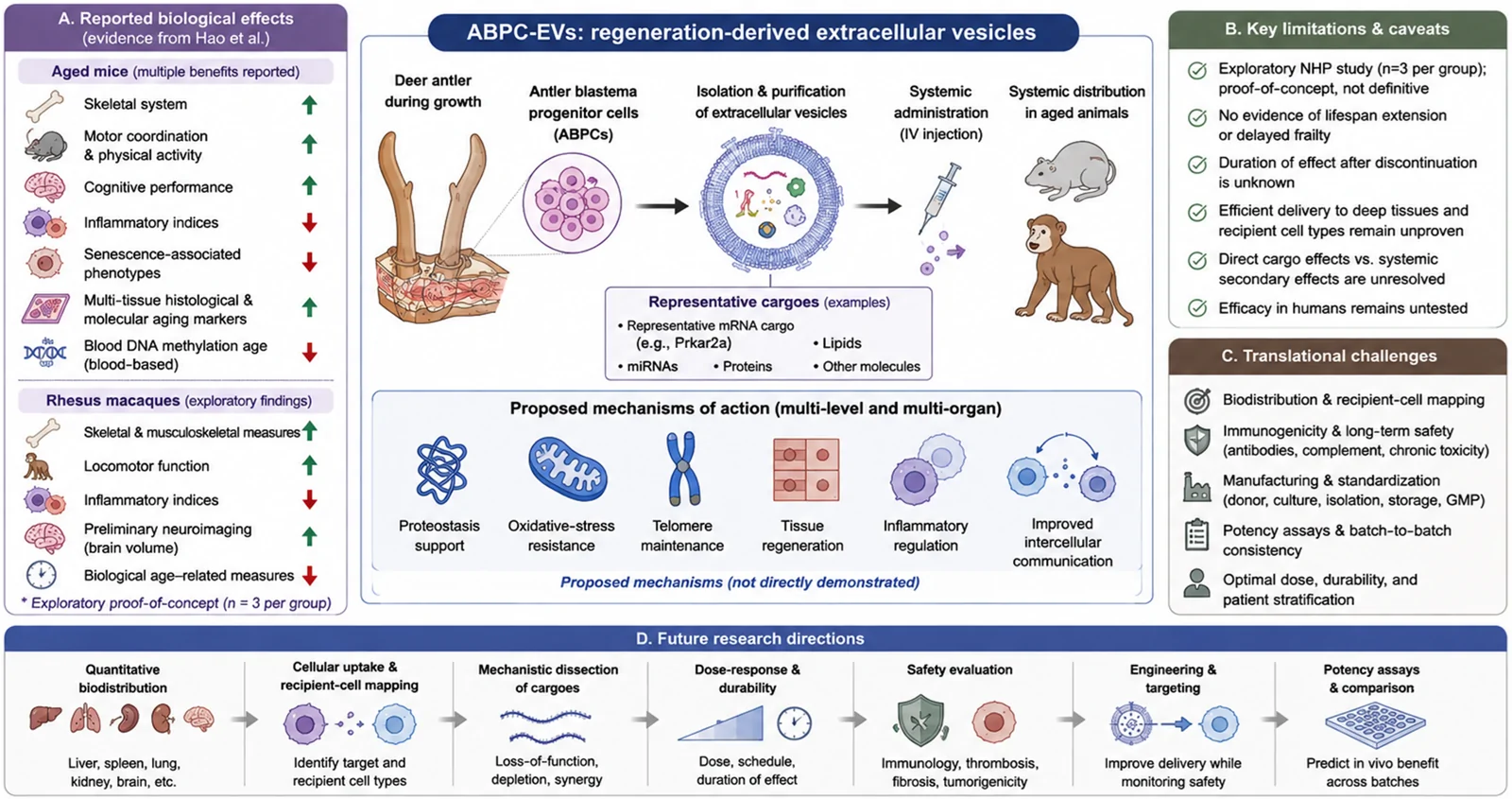
** Antler blastema progenitor cell-derived extracellular vesicles (ABPC-EVs) attenuate multiple aging-associated phenotypes.** Annual antler regeneration is sustained by highly proliferative ABPCs, which are distinct from conventional bone marrow-derived mesenchymal stromal cells. ABPC-EVs contain diverse RNA and protein cargoes associated with regenerative and stress-response pathways. Prkar2a mRNA was experimentally implicated in anti-senescence and osteogenic effects, whereas the contribution of PRKAR2A protein and other proposed downstream pathways requires further validation. In aged mice, systemic ABPC-EV administration improved selected skeletal, motor, cognitive, inflammatory, and senescence-associated measures. In exploratory rhesus macaque experiments (n = 3 per group), treatment produced promising but preliminary functional, imaging, inflammatory, and biological-age-related changes. ABPC-EVs reduced blood DNA methylation age while attenuating aging-associated histological and molecular alterations in multiple tissues; they did not demonstrate complete reversal of organismal aging or efficacy in humans. Translation will require quantitative biodistribution, long-term immunological and oncological safety assessment, standardized serum-free and GMP-compatible manufacturing, potency testing, and validated product-release criteria. Potential human applications remain to be established.
